# Neuroblastoma: A Case of Massive Hepatomegaly

**DOI:** 10.7759/cureus.16731

**Published:** 2021-07-29

**Authors:** Qamar Ali, Muhammad Ibraiz Bilal, Fawwad A Ansari, Muhammad Umer Riaz Gondal, Adnan Arif

**Affiliations:** 1 Paediatrics, Shifa International Hospital Islamabad, Islamabad, PAK; 2 Internal Medicine, Shifa International Hospital Islamabad, Islamabad, PAK; 3 Diagnostic Radiology, Shifa International Hospital Islamabad, Islamabad, PAK

**Keywords:** neuroblastoma, hepatomegaly, prognosis, management, chemotherapy

## Abstract

Neuroblastoma is the most common embryonal tumor of childhood and has a variable presentation. Stage 4S neuroblastoma, described as a localized primary tumor in an infant with metastasis to skin, liver, or bone marrow, is an exception to the poor prognosis seen in widespread metastasis of neuroblastoma. Survival in infants with this stage of the disease is over 90%. Stage 4S with massive liver involvement, however, confers a poor prognosis. We need more research on the optimum treatment modality for patients with Stage 4S disease and massive hepatomegaly to improve patient outcomes.

## Introduction

Neuroblastoma is an embryonic tumor of the peripheral sympathetic nervous system [1]. It is one of the most common extra-cranial solid tumors in children, comprising 8-10% of all childhood tumors [2]. This disease exhibits unique features, such as early age of onset, high frequency of metastatic disease at diagnosis in patients over one year of age, and the tendency for spontaneous regression of tumors in infants [3]. The median age of diagnosis is 19 months [1]. Multiple factors determine a patient’s clinical presentation including, tumor location, size, degree of invasion, effects from catecholamine secretion, and symptoms due to paraneoplastic syndromes [2]. Metastasis can occur by hematogenous or lymphatic route, seeding bone marrow, liver, and bone [2]. Diagnosis of neuroblastoma is made based on histologic confirmation combined with chemical profiling (for example, urinary vanillylmandelic acid) and imaging characteristics (suprarenal mass on imaging) [1]. The mainstay of treatment comprises chemotherapy, surgical resection, or radiotherapy [2]. We report a case of a three-month-old infant who presented with neuroblastoma with massive hepatomegaly.

## Case presentation

A three-month-old male infant, previously healthy, presented to us with significant abdominal distension for three weeks. There was a complaint of watery diarrhea not associated with blood or vomiting. The patient had no complaints of fever, nausea, vomiting, or food intolerance. The baby was born by caesarian section at term, and there were no complications. Immunizations at the presentation were up to date. The patient had a normal development of age-related milestones. Family history was not significant for any malignancies or metabolic disorders.

On examination, the patient was hemodynamically stable. There was no observed jaundice or any significant skin marks. On inspection, his abdomen was grossly distended with massive hepatomegaly. Bowel sounds were audible. Auscultation revealed bilateral clear air entry and did not reveal any murmurs.

**Figure 1:**
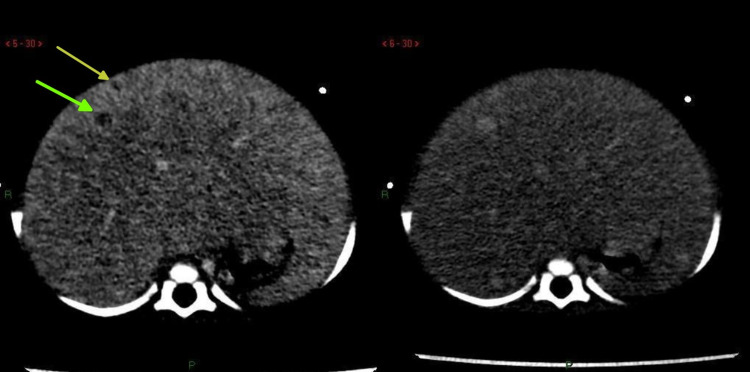
Portal venous phase (left) shows predominant hypodense nodules with a rim of peripheral enhancement (arrows) with gradual central fill on corresponding equilibrium phase sequence (right).

Blood tests done on the patient showed a high white blood cell count (27,270 cells/mm^3^) with 58% percent lymphocytes and a hemoglobin of 9.8 g/dL. Liver function tests showed an increased gamma GT of 116 IU/L with total and direct bilirubin in the normal range. The patient also had increased alpha-fetoprotein (92.7 ng/mL) and lactate dehydrogenase (LDH) levels (251 U/L). The triglyceride level was high (156 mg/dL) but the cholesterol level was normal. Ultrasound abdomen confirmed massive hepatomegaly with ill-defined heterogeneous areas compressing and displacing surrounding structures. A right suprarenal echogenic mass was seen as well. A contrast-enhanced CT scan of the abdomen done showed gross hepatomegaly. The lower margin of the liver was seen reaching up to the right iliac fossa and its left margin, crossing the midline and reaching the left hypochondrium. Small-sized hypodensities were seen in the liver, with the largest one in the right hepatic lobe measuring approximately 6 mm in size. CT liver dynamic (Figure 1) performed showed innumerable hypodense nodules on arterial and venous phase, with hyperdense lesions on delayed phase, findings consistent with a diagnosis of infantile hepatic hemangioendothelioma.

**Figure 2:**
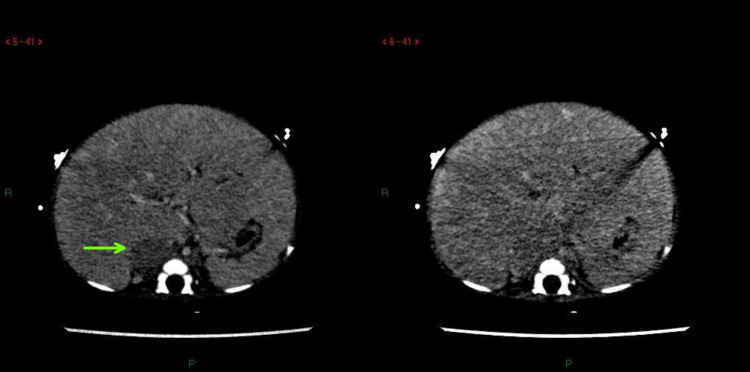
Right adrenal mass appearing predominantly hypodense on venous phase (left) with gradual peripheral enhancement on equilibrium phase images (right).

However, a large rounded soft tissue density nodule was seen in the right suprarenal location showing gradual enhancement and raising suspicion for neuroblastoma (Figure 2).

Differential diagnosis of infantile hemangioendothelioma and neuroblastoma was considered based on imaging findings. Subsequently, 24-hour urinary vanillyl-mandelic acid levels were done, which turned out to be high (13.1), favoring the likelihood of neuroblastoma.

**Figure 3:**
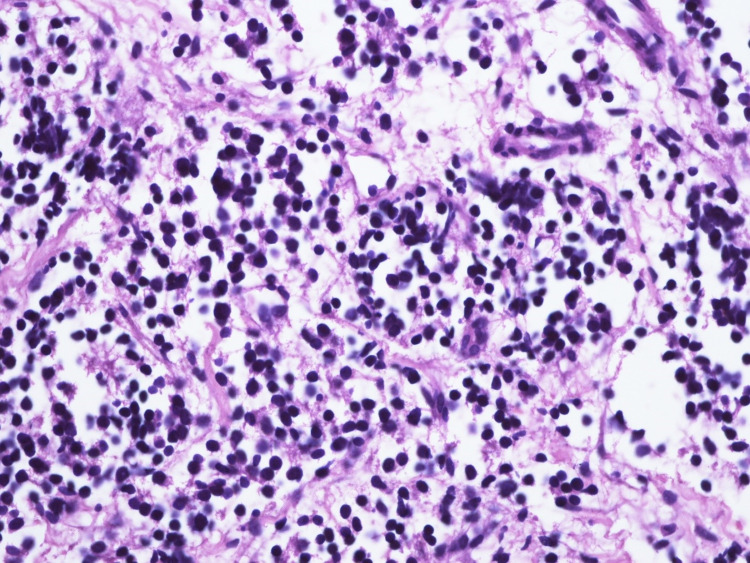
Liver biopsy specimen showing small, round blue cells.

**Figure 4:**
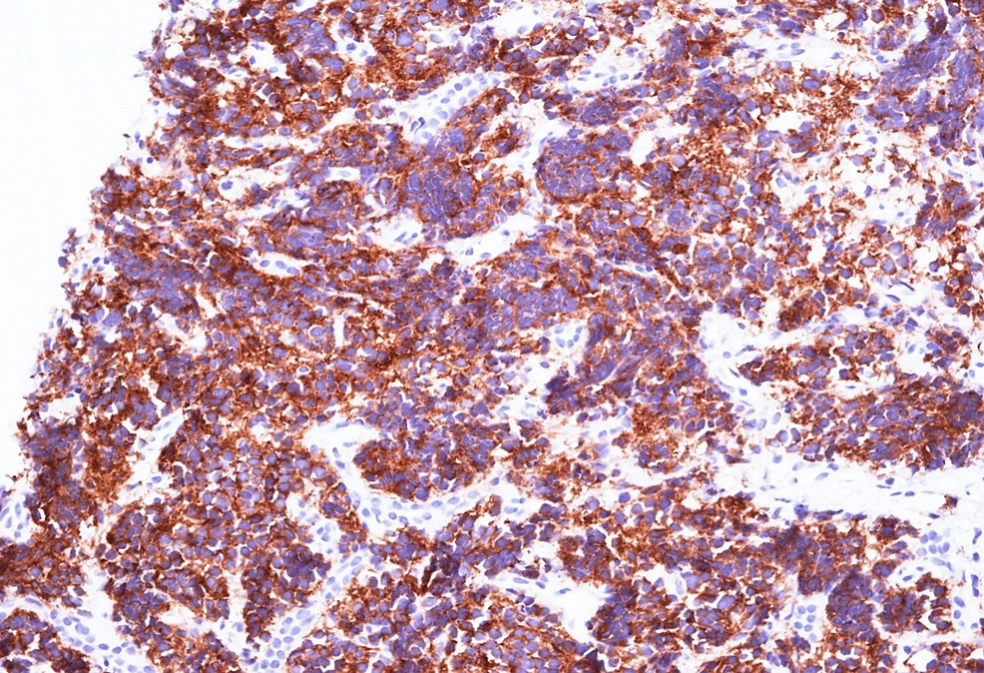
Immunohistochemistry of biopsy specimen showing synaptophysin positivity.

Ultrasound-guided biopsy of the liver revealed a tumor with small, round blue cells (Figure 3), and immunohistochemistry was positive for synaptophysin, confirming the diagnosis of infantile neuroblastoma (Figure 4).

After discussion with the pediatric oncology department at our tertiary care hospital, the patient was admitted to the hospital for chemotherapy and underwent one session of chemotherapy with etoposide and carboplatin. Unfortunately, after discharge, the patient was lost to follow up. Upon contacting the family, we discovered that the patient had developed fever 10 days after the chemotherapy. In a local outside hospital, the patient was diagnosed with febrile neutropenia and passed away secondary to sepsis.

## Discussion

Neuroblastoma is the most common cancer in infancy. Ninety percent of patients are less than five years of age at diagnosis [4]. The manifestations of neuroblastoma are variable depending on the anatomical location of the tumor and the presence of paraneoplastic syndromes. Approximately 46% of neuroblastomas arise from the adrenal gland, 18% from an extra-adrenal abdominal location, and the remainder from the thorax, neck, pelvis, and other locations [1]. Metastasis to regional lymph nodes, bone marrow, cortical bone, liver, and skin is common [5].

Prognosis depends on factors such as patient age, size of the tumor, stage of disease, and biological characteristics [6]. Young children beyond the newborn age appear to have the best prognosis, particularly until 18 months [7].

The International Neuroblastoma Staging System (INSS) is the most common staging system used for neuroblastoma. In this system, Stage 4S is defined as the localized primary tumor, with dissemination to the skin, liver, or bone marrow (limited to infants < 1 year of age) [8]. This staging category is an exception to the typically poor prognosis for children with widespread metastases from neuroblastoma. Survival for infants in this category is over 90% [9].

It is noteworthy that symptomatic hepatomegaly in Stage 4S neuroblastoma is a rare finding [10]. Although Stage 4S neuroblastoma is a unique metastatic cancer that confers a favorable outcome, the outcome is not favorable in the subset of patients who present with massive liver involvement, as is the case in our patient.

A study showed that out of eight people who had 4S neuroblastoma with massive liver involvement, one patient did not require any intervention while the disease progressed in the remaining seven. Out of these seven patients, three patients responded to chemotherapy [11]. Another study suggested that we can improve the outcome for 4S neuroblastoma with pre-emptive chemotherapy for growing hepatomegaly [12]. Considering this infirm evidence and absence of explicit guidelines, we started our patient on chemotherapeutics. Our patient responded poorly to the chemotherapy and he could not tolerate the therapeutic regimen. He developed neutropenic fever after the first session of chemotherapy and unfortunately did not survive.

Whether the patient would have been better off with conservative management alone or alternative treatment with radiotherapy remains a question. It is a medical dilemma that requires risk and benefit assessment before any of these options is considered.

## Conclusions

Massive hepatomegaly in Stage 4S neuroblastoma is a relatively rare entity and signals a grave prognosis. There is no clear-cut data available in the literature regarding the treatment of such cases. We based the management on the judgment of the treating physicians, and there is no consensus. We need more studies on this comparatively unique subset of neuroblastoma patients, and a well-defined treatment outline needs to be devised to allow a much more unified approach for its management in the future.
